# Can a mechanical circulatory support comprehensive approach to cardiogenic shock at referral centers reduce 30-day mortality?

**DOI:** 10.3389/fcvm.2024.1509162

**Published:** 2025-01-16

**Authors:** Marina Pieri, Mario Iannaccone, Francesco Burzotta, Giulia Botti, Cristina Aurigemma, Carlo Trani, Silvia Ajello, Savino Altizio, Tommaso Sanna, Enrico Romagnoli, Lazzaro Paraggio, Luigi Cappannoli, Anna Mara Scandroglio, Alaide Chieffo

**Affiliations:** ^1^ Department of Anesthesia and Intensive Care, IRCCS San Raffaele Scientific Institute, Milan, Italy; ^2^ School of Medicine, Vita-Salute San Raffaele University, Milan, Italy; ^3^ Department of Cardiology, San Giovanni Bosco Hospital, ASL Città di Torino, Turin, Italy; ^4^ School of Medicine, Università Cattolica del Sacro Cuore, Rome, Italy; ^5^ Department of Cardiovascular Sciences, Fondazione Policlinico Universitario Agostino Gemelli IRCCS, Università Cattolica del Sacro Cuore, Rome, Italy; ^6^ Interventional Cardiology Unit, IRCCS San Raffaele Scientific Institute, Milan, Italy; ^7^ Dipartimento CUORE, Fondazione Policlinico Univeristario A. Gemelli IRCCS Roma, Rome, Italy

**Keywords:** cardiogenic shock, mortality, risk score, mechanical circulatory support, Impella, inotropes

## Abstract

Although mortality risk prediction in cardiogenic shock (CS) is possible, assessing the impact of the multitude of therapeutic efforts on outcomes is not straightforward. We assessed whether a temporary mechanical circulatory support comprehensive approach to the treatment of CS may reduce 30-day mortality as compared to expected mortality predicted by the recently proposed Cardiogenic Shock Score (CSS). Consecutive CS patients supported by pVAD Impella (Abiomed, Danvers, MA) at two national referral centers were included. 170 patients were included: age was 65 ± 13 years, and 75.9% were male and acute myocardial infarction was the prevalent cause of shock (71.1%). Expected mortality according to CSS was higher than observed (51.8% vs. 41.5%, *p* < 0.001), this trend being particularly evident for CSS > 4. The AUC ROC curve confirmed poor diagnostic accuracy in this population (AUC 0.53 CI: 0.23–0.82, *p* = 0.83). The lower observed mortality compared to the expected mortality in critical cardiogenic shock population underscores the role of a comprehensive approach to acute cardiac care patients at referral centers, which should consider including temporary mechanical circulatory support.

## Introduction

Despite significant advancements in the treatment of cardiogenic shock (CS), including mechanical circulatory support, its mortality has remained high (1). Therefore, the stratification of patients' mortality risk is of great clinical interest to guarantee to each patient the most appropriate type and timing of treatments (2). Recently, the novel Cardiogenic Shock Score (CSS) has emerged as a powerful and easily implemented tool (3) to predict the outcome of patients irrespective of the cause of CS and the type of treatment received, showing superior predictive ability compared to established scores (4, 5). Although CS outcome prediction is possible, assessing the impact of the multitude of therapeutic efforts on outcomes remains challenging in critically ill patients.

The present study aimed to assess whether a mechanical circulatory support comprehensive approach to the treatment of CS with percutaneous ventricular assist devices (pVAD) may reduce 30-day mortality compared to the expected mortality predicted by the recently proposed CSS.

## Methods

Consecutive patients with CS (6) treated with Impella 2.5, Impella CP, Impella 5.0, or Impella RP in IRCCS San Raffaele Scientific Institute, Milan, Italy and Institute of Cardiology and Fondazione Policlinico Universitario A. Gemelli IRCCS, Rome; Italy from 2013 to 2018 were included. Briefly, data related to medical history, procedural characteristics, 30-day and one-year outcomes were collected from each centre and included in a pre-specified structured data set. Adverse events were then adjudicated by two independent cardiologists using source documents provided by each center. The collection of data at each participating site was performed according to the policies of the local institutional review board/ethics committee.

The primary objective of the study was to assess the effect of a comprehensive approach to cardiogenic shock at referral centers, encompassing temporary mechanical circulatory support (tMCS), on 30-day mortality risk, as assessed by the novel CSS. In addition, the composite of all-cause death, rehospitalization for heart failure, left ventricle assist device implantation, or heart transplantation (HT), overall referred to as major adverse cardiac events (MACE) was evaluated at 1 year.

Categorical variables are reported as counts and percentages, whereas continuous variables as mean and standard deviation or median and interquartile range (IQR). Gaussian or non-Gaussian distribution was evaluated with the Kolmogorov-Smirnov test. The *t*-test was used to assess differences between normally distributed continuous variables, paired or unpaired according to the tested variable, the Mann-Whitney *U* test for non-Gaussian variables, the *χ*^2^ test for categorical variables (expected vs. observed mortality), and Fisher exact test for 2 × 2 tables. The distribution of the population and predicted and observed mortality within risk categories were calculated and evaluated by XY correlation.

The discriminative ability of the risk prediction model was assessed by the area under the receiver operating characteristic (ROC) curve (AUC) or c-statistic. A two-sided *p*-value <0.05 was regarded as statistically significant. Analyses were performed with SPSS® Statistics v24 and STATA v17 (StataCorp, College Station, Texas).

## Results

One hundred and seventy patients were included in the analysis: the mean age was 65 ± 13 years, and 75.9% were male. Patients' characteristics are shown in Table 1. Acute myocardial infarction was the prevalent cause of shock, accounting for 71.1% of cases. Mean arterial pressure at presentation at implantation was 64.9 ± 19.9 mmHg, mean heart rate was 95 ± 25 bpm. Mean lactates were 6.6 ± 5.4 mg/dl, mean baseline creatinine was 1.6 ± 1.3 mg/dl and mean blood glucose was 215.3 ± 91.8 mg/dl.

**Table 1:** Patients’ characteristics.

Parameter	Value
Baseline characteristics	
Age, years	65.3 ± 13.0
Male gender, *n*	129 (75.9)
BMI	26.2 ± 4.6
Smoking, *n*	68 (40.0)
Hypertension, *n*	76 (44.7)
Dyslipidemia, *n*	62 (36.5)
Diabetes Mellitus, *n*	47 (27.6)
Prior Myocardial infarction, *n*	52 (30.6)
Prior TIA or Stroke, *n*	9 (5.3)
Prior PCI, *n*	54 (31.8)
Prior CABG, *n*	9 (5.3)
PAD, *n*	21 (12.4)
Chronic heart failure, *n*	45 (26.5)
Atrial fibrillation, *n*	16 (9.4)
Chronic Kidney Disease, *n*	33 (19.4)
Baseline Lactates, mmol/L	6.6 ± 5.4
Lactates >2 mmol/L, *n*	106 (62.4)
Baseline Creatinine, mg/dl	1.6 ± 1.3
Baseline Haemoglobin, g/dl	12.2 ± 2.3
Glucose level at presentation, mg/dl	215.3 ± 91.8
Ejection Fraction, %	23.7 ± 12.1
Heart Rate, bpm	95 ± 25
MAP, mmHg	64.9 ± 19.9
Inotropic therapy, *n*	131 (77.1)
>1 inotrope, *n*	101 (59.4)
Mechanical Ventilation, *n*	132 (77.6)
Etiology of Cardiogenic Shock	
STEMI, *n*	90 (52.9)
NSTEMI, *n*	31 (18.2)
Acute Myocarditis, *n*	10 (5.8)
VT Ablation, *n*	9 (5.3)
Other, n	30 (17.8)
Other MCS support	
IABP, *n*	66 (38.8)
ECMO, *n*	58 (34.1)
Treatment Escalation	
ECMO, *n*	24 (14.1)
VAD, *n*	10 (5.8)
Heart Transplantation, *n*	2 (1.2)
ECMO + VAD, *n*	5 (2.9)
ECMO + VAD + Heart Transplantation, *n*	3 (1.8)
CCS Score	
≤4	44 (25.8)
5–8	81 (47.6)
≥9	45 (26.5)

Values are expressed as number (percentage) or mean ± standard deviation.

BMI, body mass index; TIA, transient ischemic attack; PCI, percutaneous coronary intervention; CABG, coronary artery bypass graft; PAD, peripheral artery disease; MAP, mean arterial pressure; OHCA, out-of-hospital cardiac arrest; CPR, cardiopulmonary resuscitation; STEMI, ST-elevation myocardial infarction; NSTEMI, non-ST-Elevation myocardial infarction; VT, ventricular tachycardia; IABP, intra-aortic balloon pump; ECMO, extracorporeal membrane oxygenator; VAD, ventricular assist device.

The prevalent etiology of CS was ischemic due to acute ST-elevation myocardial infarction 52.9%; 24.7% of the patients experienced out-of-hospital cardiac arrest and 77.6% required mechanical ventilation. Pharmacological support with catecholamines was needed in 77.1% of patients. Cardiopulmonary resuscitation (CPR) was rapidly effective (<30 min) in 20% of patients, whereas 6% required extensive CPR (>30 min) and 14.7% experienced refractory cardiac arrest. Mean duration of Impella support was 96 ± 154 h, 34.1% of patients were previously or concomitantly supported with veno-arterial extracorporeal membrane oxygenation (VA ECMO), 38.8% patients received intra-aortic balloon pump before Impella support, and 59.4% were treated with more than 1 inotrope. The most used device was Impella 2.5, in 66.5% of cases. After the implantation of the Impella device, escalation of mechanical circulatory support or heart transplantation was performed in one quarter of patients (24 patients were upgraded to VA ECMO support, 10 patients eventually received a durable left ventricular assist device, 2 patients underwent cardiac transplantation, and 8 patients required a combination of advanced support techniques).

**Figure 1:**
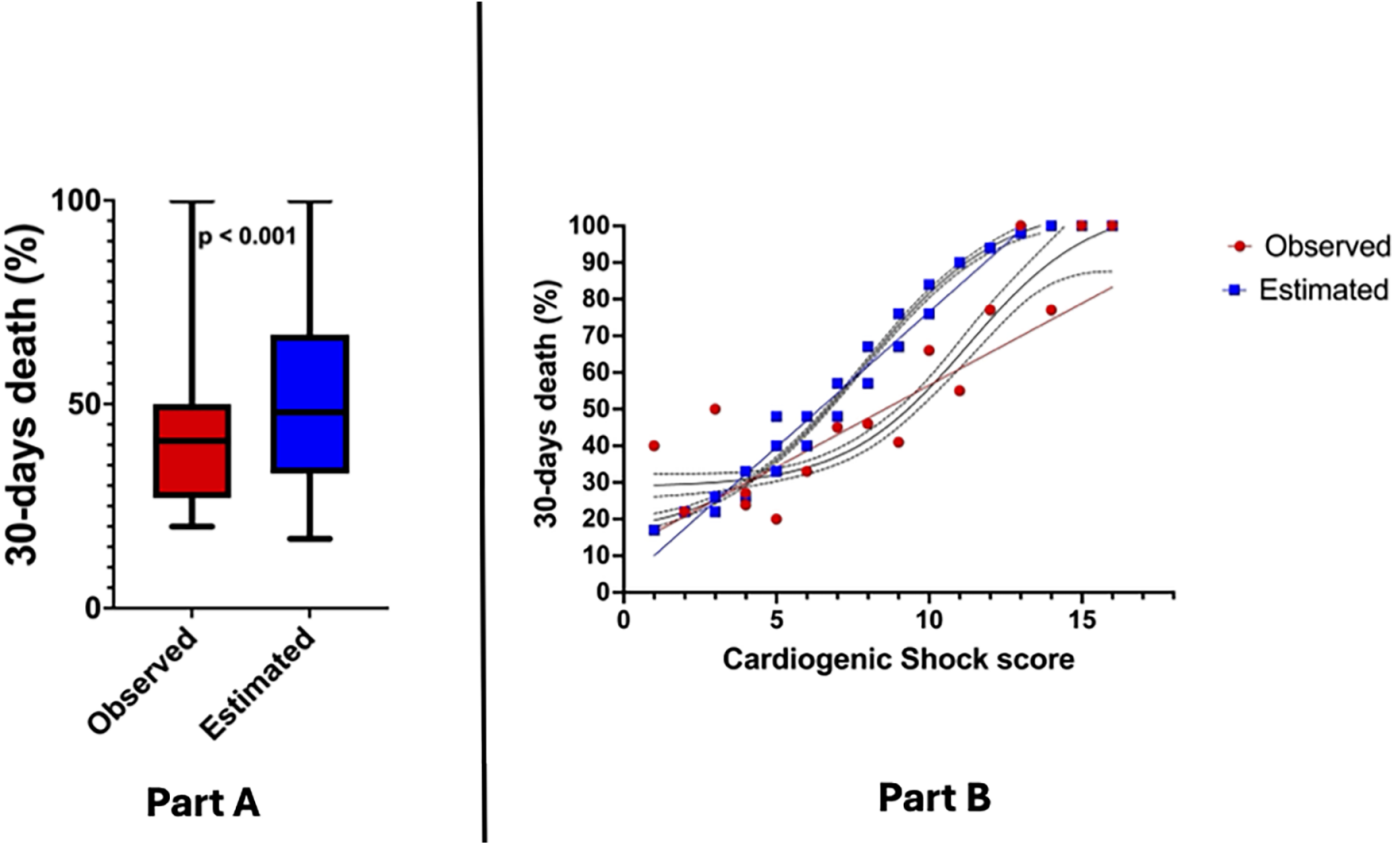
**(Part A)** Observed vs. expected 30-day mortality in study population; **(Part B)** association between cardiogenic shock score and 30-day mortality.

Regarding the calculation of the CCS score, the population was distributed as follows: 25.8% of the patients had a CSS ≤ 4, 47.6% scored between 5 and 8, and 26.5% of patients scored ≥ 9. Expected 30-day mortality according to CSS was higher than observed (51.8% vs. 41.5%, *p* < 0.001—Figure 1A), this trend being particularly evident for score values > 4 (Figure 1B). The AUC ROC curve confirmed poor diagnostic accuracy in this population (AUC 0.53 CI: 0.23–0.82, *p* = 0.83).

## Discussion

Our findings draw attention on the relevance of the approach to CS by clinicians and the need to quantify the impact of the implemented therapeutic efforts on mortality risk. The principal finding of our study was that we observed a statistically significant reduction of recorded mortality compared to what was expected according to CSS. Such results in the authors' opinion should be ascribed to the following elements: patients were treated at national referral centers for CS; management of CS patients oversaw a multidisciplinary shock team; a comprehensive approach to CS with tMCS support was adopted. Our findings are in line with existing literature showing improved outcomes in high-volume shock centers (7, 8), where patients are treated according to dedicated shock protocols and shock teams (9), and with a robust MCS program in place (10). With reference to MCS therapy, on one hand the association of hospital volume with outcome is not a new finding (11, 12), given that in the present year for the first time a randomized controlled trial has also shown significant survival benefits in a population of acute myocardial infarction CS patients treated with microaxial flow pumps (13) compared to standard therapy, further corroborating our findings. On top of this, our study presented an innovative approach (i.e., comparing observed vs. expected mortality risk with a robust statical analysis) that may help to document and quantify the impact on major clinical outcomes in a context of lack or biased randomized control trial due to logistic and ethical reasons. The choice of CSS among the multiple existing mortality risk prediction in CS (above all IABP-SHOCK II and CardShock risk scores (4, 5) was dictated by several considerations. First, CSS was developed and validated in large populations of patients compared to the other two scores. Furthemore, CSS was developed on a mixed population of CS with a 1:1 ratio between AMI related and non ischemic CS: this makes the CSS particularly valid to test mortality risk in all real clinical life CS patients. Although the majority of the patients of the present study had AMI related CS, making them suitable also for mortality risk prediction with CardShock score (developed in a population with >80% AMI CS incidence) (5) and IABP-SHOCK II (developed only in AMI CS patients) (4), we favoured CSS because he shows superior predictive ability when directly compared to IABP-SHOCK II and CardShock scores (3). Finally, CSS includes simple, quick and easy-to-collect at bedside parameters: since it does not require coronary angiography data, it can be calculated immediately upon patient admission, thus providing prompt information that may guide early clinical management of critically ill patients. It would also be relevant from a clinical point of view to assess wether the reduction in mortality observed in the present study is confirmed also with different strategies of MCS, including VA ECMO and IABP. While literature comparing different MCS approaches in CS is growing (14, 15), it is difficult to identify large homogenous patients'population for comparison and the present study itself was not powered to assess this outcome. We also acknowledge the limitations of our approach, that might have influenced the results, especially the limited sample size, the bicentric rather then multicentric approach and the lack of a comparison group not receiving MCS. Indeed, since all patients received mAFP support, discriminating if the survival benefit was due to MCS, to the referral center or to the team is not straightforward. Furthermore, we are aware that the complexity of critically ill CS patients is only partially captured by a score, even if the most valid available, and that this may at least partially affect results. Finally, in the era of DangerShock Trial, we have learnt that complications in MCS patients are frequent, with possible negative implications on outcomes (13). The burden of complications in MCS patients therefore makes the applicability of predictive models more complex.

In conclusion, the main result of this multicenter study was that the mortality rate observed in a population of critically ill CS patients admitted to national referral centers for CS with a dedicated multidisciplinary shock team using a comprehensive approach to the treatment of CS, encompassing temporary mechanical circulatory support with pVAD, was lower than expected according to the CSS, a well-established prognostic score in this field.

## Data Availability

As per institutional policy dataset will be made available upon motivated request to the corresponding author. Requests to access these datasets should be directed to pieri.marina@hsr.it.

## References

[1] Tehrani BN Truesdell AG Psotka MA Rosner C Singh R Sinha SS A standardized and comprehensive approach to the management of cardiogenic shock. JACC Heart Fail. (2020) 8(11):879–91. 10.1016/j.jchf.2020.09.005 33121700 PMC8167900

[2] Kapur NK Kanwar M Sinha SS Thayer KL Garan AR Hernandez-Montfort J Criteria for defining stages of cardiogenic shock severity. J Am Coll Cardiol. (2022) 80(3):185–98. 10.1016/j.jacc.2022.04.049 35835491

[3] Beer BN Jentzer JC Weimann J Dabboura S Yan I Sundermeyer J Early risk stratification in patients with cardiogenic shock irrespective of the underlying cause—the cardiogenic shock score. Eur J Heart Fail. (2022) 24(4):657–67. 10.1002/ejhf.2449 35119176

[4] Pöss J Köster J Fuernau G Eitel I de Waha S Ouarrak T Risk stratification for patients in cardiogenic shock after acute myocardial infarction. J Am Coll Cardiol. (2017) 69:1913–20; 22. 10.1016/j.jacc.2017.02.027 28408020

[5] Harjola VP Lassus J Sionis A Køber L Tarvasmäki T Spinar J Clinical picture and risk prediction of short-term mortality in cardiogenic shock. Eur J Heart Fail. (2015) 17:501–9. 10.1002/ejhf.260 25820680

[6] Naidu SS Baran DA Jentzer JC Hollenberg SM van Diepen S Basir MB SCAI SHOCK stage classification expert consensus update: a review and incorporation of validation studies: this statement was endorsed by the American college of cardiology (ACC), American college of emergency physicians (ACEP), American heart association (AHA), European society of cardiology (ESC) association for acute cardiovascular care (ACVC), international society for heart and lung transplantation (ISHLT), society of critical care medicine (SCCM), and society of thoracic surgeons (STS) in December 2021. J Am Coll Cardiol. (2022) 79(9):933–46. 10.1016/j.jacc.2022.01.018 35115207

[7] Vallabhajosyula S Dunlay SM Barsness GW Rihal CS Holmes DR Jr. Prasad A. Hospital-level disparities in the outcomes of acute myocardial infarction with cardiogenic shock. Am J Cardiol. (2019) 124(4):491–8. 10.1016/j.amjcard.2019.05.038 31221462

[8] Chieffo A Ancona MB Burzotta F Pazzanese V Briguori C Trani C Observational multicentre registry of patients treated with IMPella mechanical circulatory support device in ITaly: the IMP-IT registry. EuroIntervention. (2020) 15(15):e1343–50. 10.4244/EIJ-D-19-00428 31422925

[9] Tehrani BN Truesdell AG Sherwood MW Desai S Tran HA Epps KC Standardized team- based care for cardiogenic shock. J Am Coll Cardiol. (2019) 73:1659–69. 10.1016/j.jacc.2018.12.084 30947919

[10] Chieffo A Dudek D Hassager C Combes A Gramegna M Halvorsen S Joint EAPCI/ACVC expert consensus document on percutaneous ventricular assist devices. Eur Heart J Acute Cardiovasc Care. (2021) 10(5):570–83. 10.1093/ehjacc/zuab015 34057173 PMC8245145

[11] Watanabe A Miyamoto Y Ueyama HA Gotanda H Jentzer JC Kapur NK Impacts of hospital volume and patient-hospital distances on outcomes of older adults receiving percutaneous microaxial ventricular assist devices for cardiogenic shock. Circ Cardiovasc Interv. (2024) 17(12):e014738. 10.1161/CIRCINTERVENTIONS.124.014738 39470586

[12] Araki T Kondo T Imaizumi T Sumita Y Nakai M Tanaka A Relationship between the volume of cases and in-hospital mortality in patients with cardiogenic shock receiving short-term mechanical circulatory support. Am Heart J. (2023) 261:109–23. 10.1016/j.ahj.2023.03.017 37031832

[13] Møller JE Engstrøm T Jensen LO Eiskjær H Mangner N Polzin A Microaxial flow pump or standard care in infarct-related cardiogenic shock. N Engl J Med. (2024) 390(15):1382–93. 10.1056/NEJMoa2312572 38587239

[14] Ardito V Sarucanian L Rognoni C Pieri M Scandroglio AM Tarricone R. Impella versus VA-ECMO for patients with cardiogenic shock: comprehensive systematic literature review and meta-analyses. J Cardiovasc Dev Dis. (2023) 10(4):158. 10.3390/jcdd10040158 37103037 PMC10142129

[15] Ahmad S Ahsan MJ Ikram S Lateef N Khan BA Tabassum S Impella versus extracorporeal membranous oxygenation (ECMO) for cardiogenic shock: a systematic review and meta-analysis. Curr Probl Cardiol. (2023) 48(1):101427. 10.1016/j.cpcardiol.2022.101427 36174742

